# Evaluation and Analysis of Outcomes of Unicondylar Knee Arthroplasty in Unicompartmental Knee Osteoarthritis

**DOI:** 10.7759/cureus.79193

**Published:** 2025-02-17

**Authors:** Manikandan N, Nabeel Thahseen A, Ragul Muruganandan, Kishore Vellingiri

**Affiliations:** 1 Department of Orthopaedics, Tirunelveli Medical College, Tirunelveli, IND; 2 Department of Orthopaedics, Vinayaka Mission’s Medical College, Karaikal, Karaikal, IND; 3 Department of Orthopaedics, Swamy Vivekananda Medical College Hospital and Research Institute, Tiruchengode, IND; 4 Department of Orthopaedics, Sri Devaraj Urs Academy of Higher Education and Research, Kolar, IND

**Keywords:** arthroplasty, knee, osteoarthritis, unicompartmental, unicondylar

## Abstract

Background

Recent increased interest in less invasive surgical techniques has led to a concurrent resurgence in unicompartmental knee arthroplasty (UKA). UKA is an effective treatment for end-stage, symptomatic unicompartmental osteoarthritis (OA) of the knee. Both clinical outcome and kinematic studies have indicated that successful UKA functions closer to a normal knee. UKA aims to restore the natural alignment of the limb, the original ligament tension, and the joint line. The aim of this study is to evaluate and analyze the outcomes of unicondylar knee arthroplasty performed in patients with unicompartmental OA of the knee joint.

Methods

This is a prospective study that includes 20 patients of unicompartmental OA knee managed with UKA in the Department of Orthopaedics, Tirunelveli Medical College, Tirunelveli, India. The implant used was JOURNEY UNI unicompartmental knee prosthesis (Smith & Nephew, Watford, England). All patients were evaluated radiologically before and after surgery. Clinical outcome was determined by using the Knee Society Scoring System (KSS) over a two-year follow-up period.

Results

According to the KSS, the average pre-operative score of 107.25 (95-119) increased to 189.30 (175-205) in the post-operative period. No intra-operative complications were noted in our study. One patient (5%) showed delayed wound healing and healed after a course of antibiotics. There were no additional procedures or revisions for implant-related complications or the occurrence of fractures.

Conclusions

This prospective study concludes that a fixed-bearing UKA provides an effective solution in treating medial compartment OA of the knee. The fixed-bearing prosthesis has the advantage that it restores the tibial and femoral joint line to the native level and at the same time corrects to previous limb alignment. This procedure also ensures effective functional recovery and pain relief. No major intraoperative or post-operative complications were observed.

## Introduction

The introduction of minimally invasive techniques has sparked significant interest in unicondylar knee arthroplasty over the past few years due to its clear benefits, such as faster recovery [1,2], lesser pain and minimal blood loss [3]. The unicompartmental knee arthroplasty (UKA) was introduced in the 1970s. The inclusion criteria set by Kozinn and Scott in 1989 have proven to enhance surgical outcomes [4]. One concern with minimally invasive techniques is that improper exposure may compromise component positioning. Unlike total knee arthroplasty (TKA), which replaces the entire knee joint, UKA involves resurfacing only the affected knee compartment while preserving the opposite compartment. UKA preserves both cruciate ligaments, resulting in a knee with nearly normal kinematics [5]. UKA also preserves bone stock in the patellofemoral joint and opposite compartment, making revision surgery easier to perform if needed [6]. Newer systems available now prioritize cutting the tibia first using extramedullary alignment guides, eliminating the thromboembolic risk associated with intramedullary guidance [7]. The position of the femoral component is then decided by the tibial cut instead of using intramedullary femoral guidance.

For certain individuals with osteoarthritis (OA) who have unicompartmental involvement, UKA presents an appealing substitute for proximal tibial osteotomy or TKA. Compared to osteotomy, it ought to have a better initial success rate and fewer complications [8-10]. There are several determinations of success after tricompartmental arthroplasty, including patient selection, surgical technique, implant design and quality of polyethylene. Flaws or imprecision in any of these may compromise the results. In several long-term series, 10- to 15-year implant survival was 93% to 96%, with good and excellent results in more than 90% of patients at the most recent follow-up [1]. Mechanical wear or loosening may be common with fixed bearing UKA, which tend to be put in looser, thereby avoiding shifting weight to the un-resurfaced compartment [11]. Component alignment, body habitus, age and activity level are also issues that can affect outcomes and should be considered when contemplating, planning and performing UKA surgery [12,13].

Although several studies have explored early outcomes, few attempts have been made to look into the diversity in functional recovery and the impact of patient-specific factors on post-operative results. The primary objective of this study is to evaluate and analyze the functional outcomes and patient-reported outcomes after UKA in patients with unicompartmental OA. Additionally, we aim to investigate and understand the relationship between pre-operative characteristics and post-operative recovery. Based on prior literature and clinical observations, we hypothesize that factors such as age, pre-operative level of function and implant alignment significantly influence the success of UKA, with younger patients and optimal implant alignment showing better functional outcomes.

## Materials and methods

This is a prospective interventional study done over a period of three years (September 2019 to September 2022) which includes a sample size of 20 patients with unicompartmental OA knee, chosen randomly and managed by UKA. The principle of surgery was to restore the natural alignment of the knee joint, the original ligament tension and the joint line. The study population comprises patients with unicompartmental OA knee attending our Outpatient Department in the Department of Orthopaedics, Tirunelveli Medical College Hospital, Tirunelveli, India. They were carefully evaluated clinically as well as radiologically and whoever fits into our inclusion criteria were chosen and managed with UKA. The implant used was a fixed-bearing JOURNEY UNI unicompartmental knee prosthesis (Smith & Nephew, Watford, England). All components were cemented. Clinical outcome was assessed by using the Knee Society Scoring System (KSS) and visual analogue scoring system (VAS). Statistical analysis includes descriptive statistics, such as mean, standard deviation and proportions using Statistical Package for the Social Sciences (IBM SPSS Statistics for Windows, IBM Corp., Version 23.0, Armonk, NY)

Inclusion and exclusion criteria

The inclusion criteria consisted of patients with isolated medial or lateral compartment disease, aged between 45 and 60 years, with Ahlbäck grade 3 or 4 deformities. Patients were required to have a minimum range of motion (ROM) of 90°, with no more than five degrees of flexion contracture and a correctable anatomical coronal deformity of up to 10° varus or 15° valgus. The opposite compartment menisci and articular cartilage must be intact, though slight fibrillation or mild chondrocalcinosis is acceptable. Patients were excluded from the study if they had symptomatic patellofemoral disease, cruciate or collateral ligament instability, decreased ROM (loss of extension >5-15° or flexion <90°), varus or valgus deformity >10°, tricompartmental disease, inflammatory arthritis (such as rheumatoid arthritis or crystalloid arthropathy) or a previous history of high tibial osteotomy.

Pre-operative evaluation

All patients were subjected to a standard radiographic review, which included anteroposterior, lateral views of the knee joint and a full-length radiograph of the lower limb to evaluate limb alignment. Additionally, standard pre-operative stress radiographs were performed. By utilizing the anterior drawer and Lachman test, as well as evaluating mediolateral stability with the knee in full extension and 30-degree flexion, all patients' knee joints were found to be stable in both the frontal and sagittal planes. We valued the patients' subjective perception that their knee was giving away.

Post-operative follow-up

The post-operative rehabilitation protocol was implemented in two phases. Phase 1 (0-3 weeks) focused on quadriceps function, ROM, and minimizing oedema, with goals of achieving full extension, flexion equal to or greater than pre-operative levels, and full weight-bearing without limping by the end of three weeks. Phase 2 (3-6 weeks) emphasized reciprocal gait on stairs, double-leg sit-to-stand without upper extremity assistance, and return to work by six weeks. Patients underwent reviews every three months following the sixth and 12th week. The last follow-up was at two years. There were no dropouts during the follow-up period. They underwent clinical evaluations and radiological assessments regarding the position of the implant, cement fixation, tibial slope, joint line height, limb alignment and loosening.

## Results

Out of 20 patients, the majority were female (n=14, 70%). The mean age of the study population was 54 years, ranging from 46 to 60 years. The right knee was more commonly affected (n=12, 60%) than the left. Most patients (n=13, 65%) presented with a pre-operative deformity of 10-degree varus (Table 1). All patients met the inclusion criteria, and there were no losses to follow-up throughout the study.

**Table 1:** Details of sample population assessed during the study

Parameter	Sample population, n=20 (100%)
Gender	
Male	6 (30%)
Female	14 (70%)
Age at Surgery	
46-50 years	2 (10%)
51-55 years	12 (60%)
56-60 years	6 (30%)
Side	
Right	12 (60%)
Left	8 (40%)
Pre-operative Varus	
8°	2 (10%)
9°	5 (25%)
10°	13 (65%)

**Figure 1:**
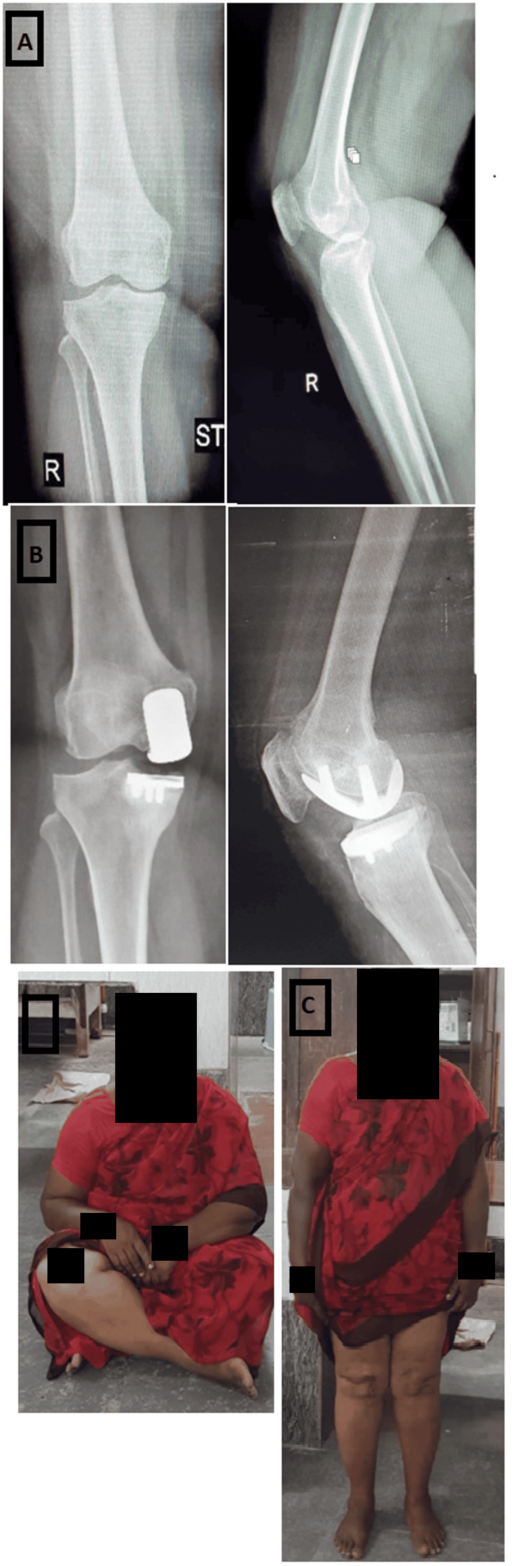
Radiographic and clinical assessment of a 50-year-old woman with severe medial knee pain (A) Pre-operative standing radiographs showing grade 3 medial osteoarthritis. The limb alignment before surgery was 10 degrees of varus. (B) Post-operative radiographs taken at a two-year follow-up. (C) The patient is now pain-free, with excellent knee function, and the radiographs show a stable and well-fixed implant. Importantly, there are no signs of progression of lateral joint space narrowing.

No intraoperative complications were noted. One patient (n=1, 5%) experienced delayed wound healing, which resolved after a course of antibiotics (Table 2). Other anticipated complications, such as infection, periprosthetic fracture, ligament rupture, polybearing or prosthesis dislocation, aseptic loosening and deep vein thrombosis, were not observed. No additional procedures or revisions were required. Interestingly, in contrast to the revision cases mentioned in the literature for mobile-bearing series [14-16], there were no recorded revisions due to bearing dislocation, instability or anterior cruciate ligament rupture. Additionally, no implant-related complications were observed during the follow-up period (Figure 1).

**Table 2:** Patients who experienced complications in their final follow-up DVT: Deep vein thrombosis; MCL: Medial collateral ligament; LCL: Lateral collateral ligament

Complications	Number of patients, n=20 (100%)
Delayed wound healing	1 (5%)
Infection	0 (0%)
Periprosthetic fracture	0 (0%)
MCL/LCL rupture	0 (0%)
Poly bearing dislocation	0 (0%)
Aseptic loosening	0 (0%)
Prosthesis dislocation	0 (0%)
DVT	0 (0%)

**Figure 2:**
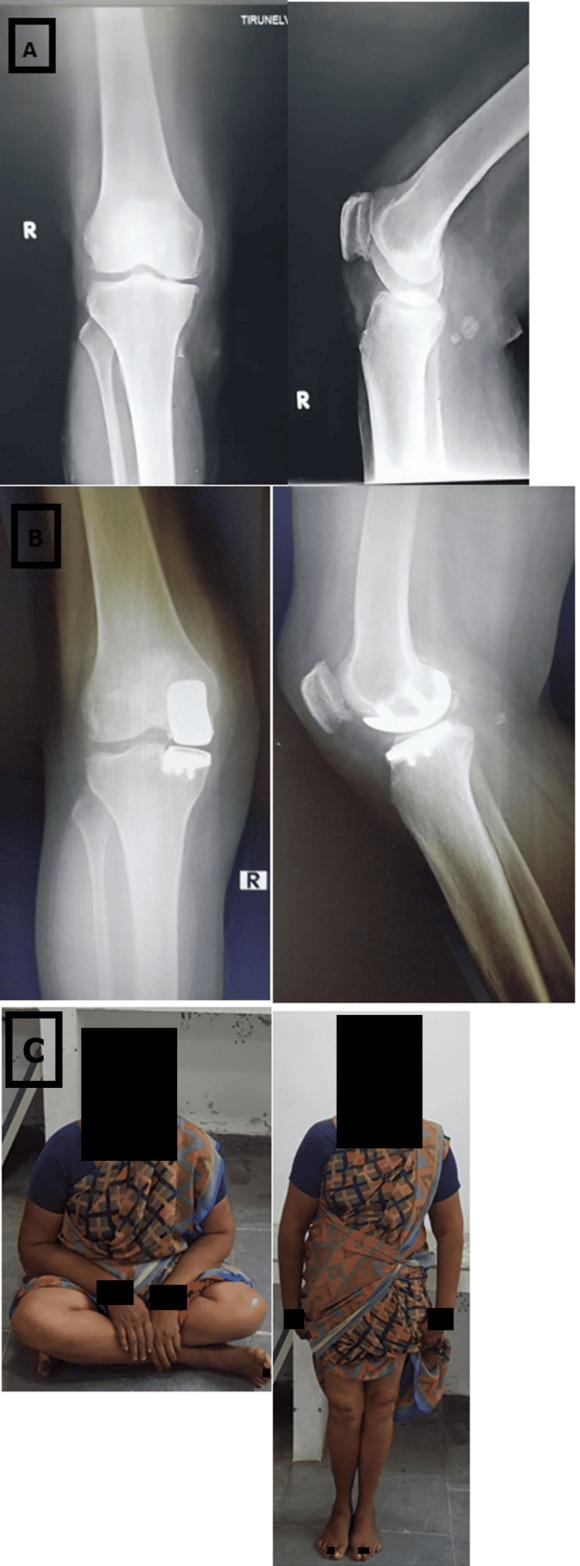
Radiographic and clinical assessment of a 58-year-old woman with severe medial knee pain (A) Pre-operative standing radiographs showing grade 3 medial osteoarthritis. The limb alignment before surgery was 9 degrees of varus. (B) Post-operative radiographs taken at a two-year follow-up. No complaint was reported by her about the knee. She could do painless stair climbing. (C) Standing radiographs show a stable and well-fixed implant and there are no signs of progression of lateral joint space narrowing.

**Figure 3:**
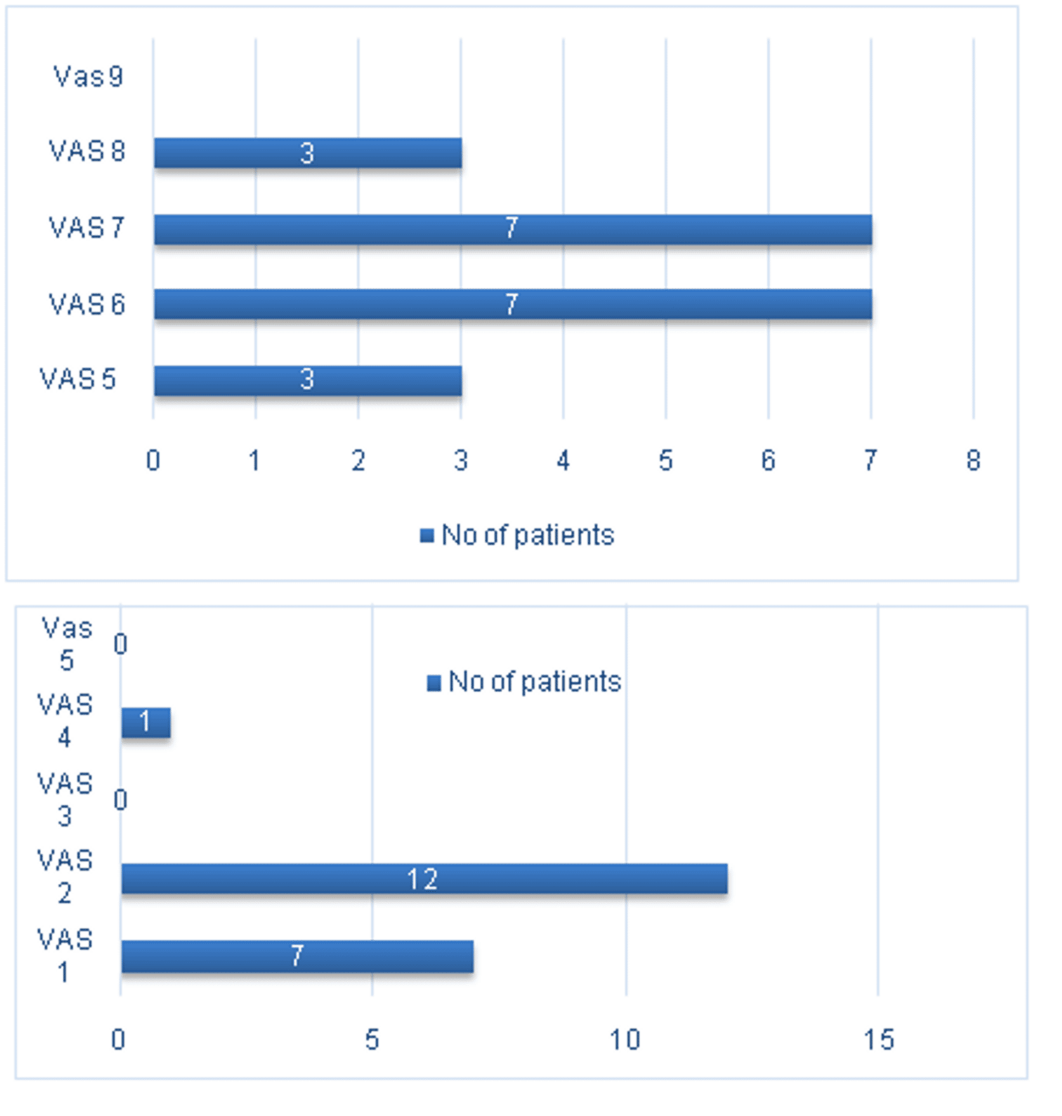
Representation of post-operative improvement of VAS scale for pain VAS: Visual analogue scale

**Figure 4:**
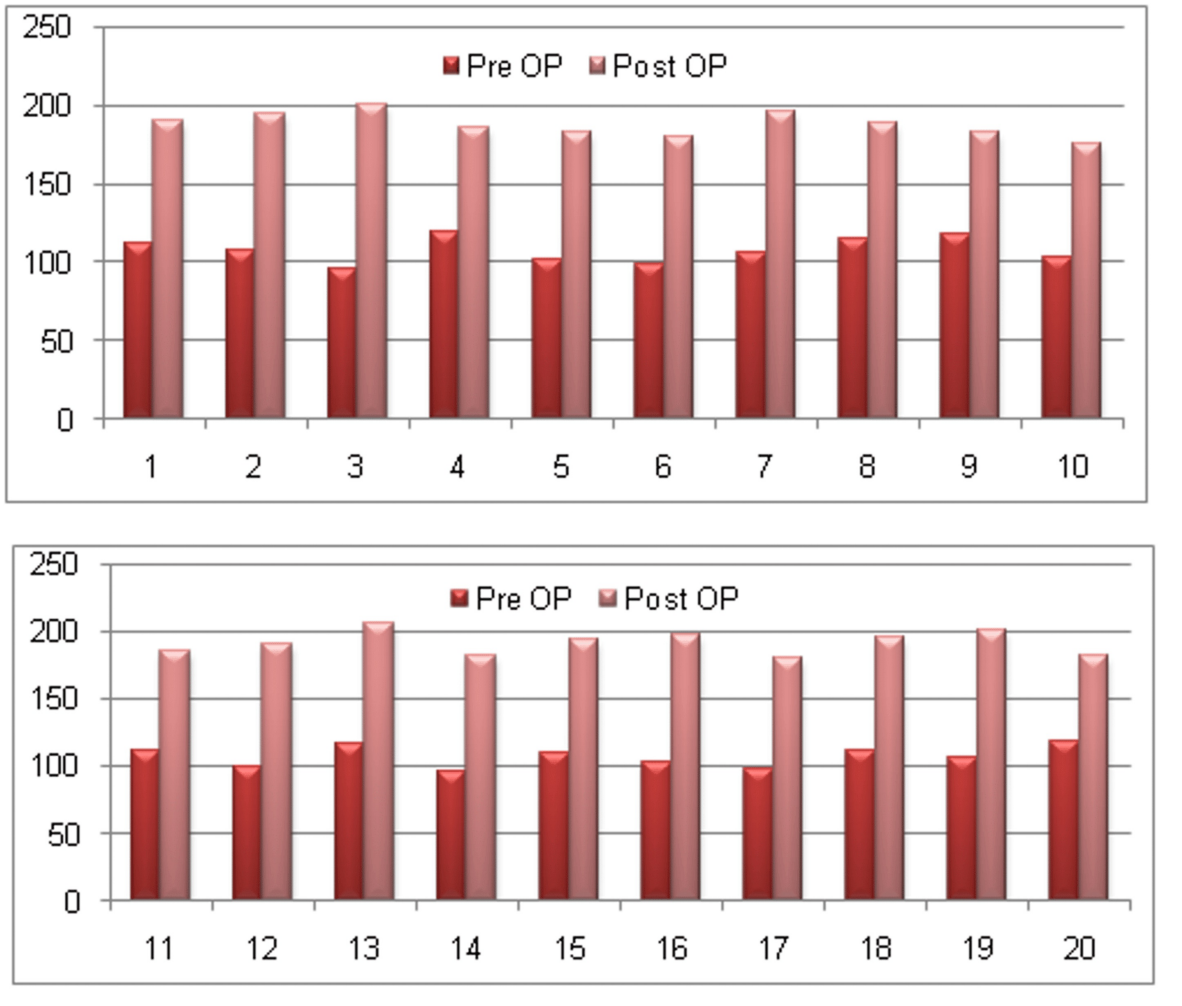
Representation of improvement in post-operative Knee Society Score (KSS) system

VAS scores for pain were analyzed in both the pre-operative and post-operative periods. The pre-operative average VAS score was 6.5 (range: 5-8), which significantly improved to a post-operative average of 1.75 (range: 1-4). The majority of patients (n=12, 60%) reported a post-operative VAS score of 2 (Figure 2), while a few (n=7, 35%) had a VAS score of 1, and one patient (n=1, 5%) had a VAS score of 4 (Figure 3). The functional outcome of the procedure was evaluated using the clinimetric quality of the KSS system. The average KSS increased from 107.25 (range: 95-119) pre-operatively to 189.3 (range: 175-205) post-operatively, with a maximum possible score of 255 (Figure 4). Mean ± SD and p-values were calculated for pre- and post-operative alignments, VAS scores and KSS scores, as shown in Table 3, with all correlations yielding a p-value of <0.0001, indicating statistical significance.

**Table 3:** Mean ± SD and p-values for pre- and post-operative alignment, VAS scores and KSS scores. The p-value is considered significant. VAS: Visual analogue scale; KSS: Knee Society Scoring system; pre-op: pre-operative; post-op: post-operative

Parameter	Pre-op (mean ± SD)	Post-op (mean ± SD)	p-value
Pre-op deformity	9.1 ± 2.02	2.4 ± 1.39	<0.0001
Pre-op VAS	6.5 ± 0.95	1.75 ± 0.72	<0.0001
Pre-op KSS	107.25 ± 7.86	189.3 ± 8.23	<0.0001

## Discussion

In cases of medial compartment OA knee, studies have revealed that mobile-bearing UKA procedures have produced positive outcomes. For example, Price and Svard's research in 2011 [14] on 682 Oxford UKAs over an average follow-up period of 5.1 years revealed survival rates of 94% and 91% at 10 and 16 years, respectively, with no available functional scores. Patients reported an American Knee Society Score (AKSS) of an average of 90 in their final follow-up, with 86% experiencing excellent or good functional outcomes [16]. No complications like component loosening or excessive poly wear were observed, and reasons for revisions included factors such as infection and lateral overload. Fixed-bearing UKAs have shown lower rates of complications like dislocation and instability [17,18]. These procedures focus only on the affected compartment of the knee, maintaining the joint line at its natural level and preserving cruciate ligaments.

Research suggests that fixed and mobile-bearing UKAs offer similar outcomes in terms of implant survival and joint function [19]. Studies have demonstrated the advantages of computer-assisted surgery in improving component positioning in total knee replacements, as well as in minimally invasive UKAs [20]. UKA procedures may be a beneficial option for certain osteoarthritic patients, providing a conservative approach that preserves ligaments and bone stock [21]. This type of replacement surgery can be particularly suitable for middle-aged patients with OA and those with a shorter life expectancy [22].

In this prospective study, we have found that the pain analysed using the VAS system was dramatically improved from an average pre-operative scale of 6.5 to an average of 1.75 post-operatively, and the operative functional knee scoring has improved very well, with the average pre-operative being 107.25, which increased to 189.3 post-operatively. The mean Oxford knee score of 43.3 points was recorded by Winnock de Grave et al. [23] at the last follow-up, with good and excellent outcomes shown by 94.6% of patients. In our series, no revisions occurred due to an increase in arthritic changes in the lateral compartment, comparable to 29% in Price and Svard [14], 48% in Pandit et al. [15] and 24% in Alnachoukati et al. [16]. Pandit et al. [15] suggested that keeping the limb alignment undercorrected thinner inserts would result in better survival rates.

This study presents several limitations. First, the size of the sample population was small. Second, even though the strict imaging protocol to obtain weight-bearing radiographs was followed, there might be small rotational variations that could possibly influence our measurements of limb alignment before and after the surgery. The follow-up period should be long enough to assess the long-term complications space narrowing and congruence alterations over time.

## Conclusions

This prospective study concludes that a fixed-bearing UKA provides an effective solution for treating medial compartment OA of the knee. The fixed-bearing prosthesis has the advantage that it restores the tibial and femoral joint line to the native level and at the same time corrects to previous limb alignment. This procedure also ensures effective functional recovery and pain relief. No major intraoperative or post-operative complications were observed.
